# Arthroscopic Modified McLaughlin Procedure and Remplissage for Treatment of Simultaneous Reverse Hill-Sachs and Hill-Sachs Lesions

**DOI:** 10.1016/j.eats.2022.03.038

**Published:** 2022-07-14

**Authors:** Santiago Arauz, David González-Martín, Marcelo Quiroga, Pedro Guillén

**Affiliations:** a Department of Orthopaedic Surgery and Traumatology, Clínica CEMTRO, Madrid, Spain; b Department of Orthopaedic Surgery and Traumatology, Hospital Universitario de Canarias, Tenerife, Spain; c Universidad de La Laguna, Tenerife, Spain

## Abstract

Hill-Sachs lesions (HSLs) can be present after a primary shoulder dislocation and may go unrecognized; this can alter the necessary bony constraint within the glenohumeral joint. To deal with HSLs, remplissage is a safe procedure with low complication rates, low recurrent instability rates, and good patient outcome scores compared with many of the other alternative techniques. On the other hand, a great number of techniques have been described to treat reverse Hill-Sachs lesions (RHSLs). In this article, we propose a method of treatment for combined simultaneous HSL and RHSL shoulder injuries. However, consensus on a specific treatment is yet to be established. We present an arthroscopic treatment guideline for patients with shoulder instability due to anterior and posterior labral lesions, HSL, and RHSL.

## Technique Video

Video Technique.

The glenohumeral joint is the most commonly dislocated joint in the body, with an overall incidence of 17 per 100,000 per year, with higher incidences in young, active individuals, especially those engaged in athletic activities.^1^^,^^2^ Posterior shoulder instability is less common than anterior shoulder instability; however, posterior instability is becoming increasingly recognized in the surgical treatment of the unstable shoulder.^3^ With advanced imaging and arthroscopic evaluation, our understanding of the injury patterns associated with instability has significantly improved.^1^

The Hill-Sachs lesion (HSL), an impaction fracture of the posterolateral humeral head, is a well-described lesion common in anterior shoulder instability. An analogous lesion is often found in posterior shoulder instability as an impaction fracture of the anterior humeral head (reverse Hill-Sachs lesion [RHSL]).^3^

To deal with HSLs, remplissage is a safe procedure with low complication rates, low recurrent instability rates, good patient outcome scores, and minimal loss of range of motion compared with many of the alternative techniques.^4^ On the other hand, a great number of techniques have been described to treat RHSLs, and consensus on a specific treatment is yet to be established.^5^ McLaughlin,^6^ in 1952, described his technique involving the transfer of the subscapularis tendon into the anterior humeral defect. Afterward, Krackhardt et al.^7^ proposed the first all-arthroscopic technique based on fixation of the subscapularis tendon into the RHSL, commonly known as “reverse remplissage.” Since then, many variations of this arthroscopic procedure have been proposed.^5^^,^^8^, ^9^, ^10^

In this article, we propose a method of treatment for combined simultaneous HSL and RHSL shoulder injuries. The purpose of this Technical Note is to show an arthroscopic treatment guideline for patients with shoulder instability due to anterior and posterior labral lesions, HSL, and RHSL.

## Surgical Technique

**Figure 1:**
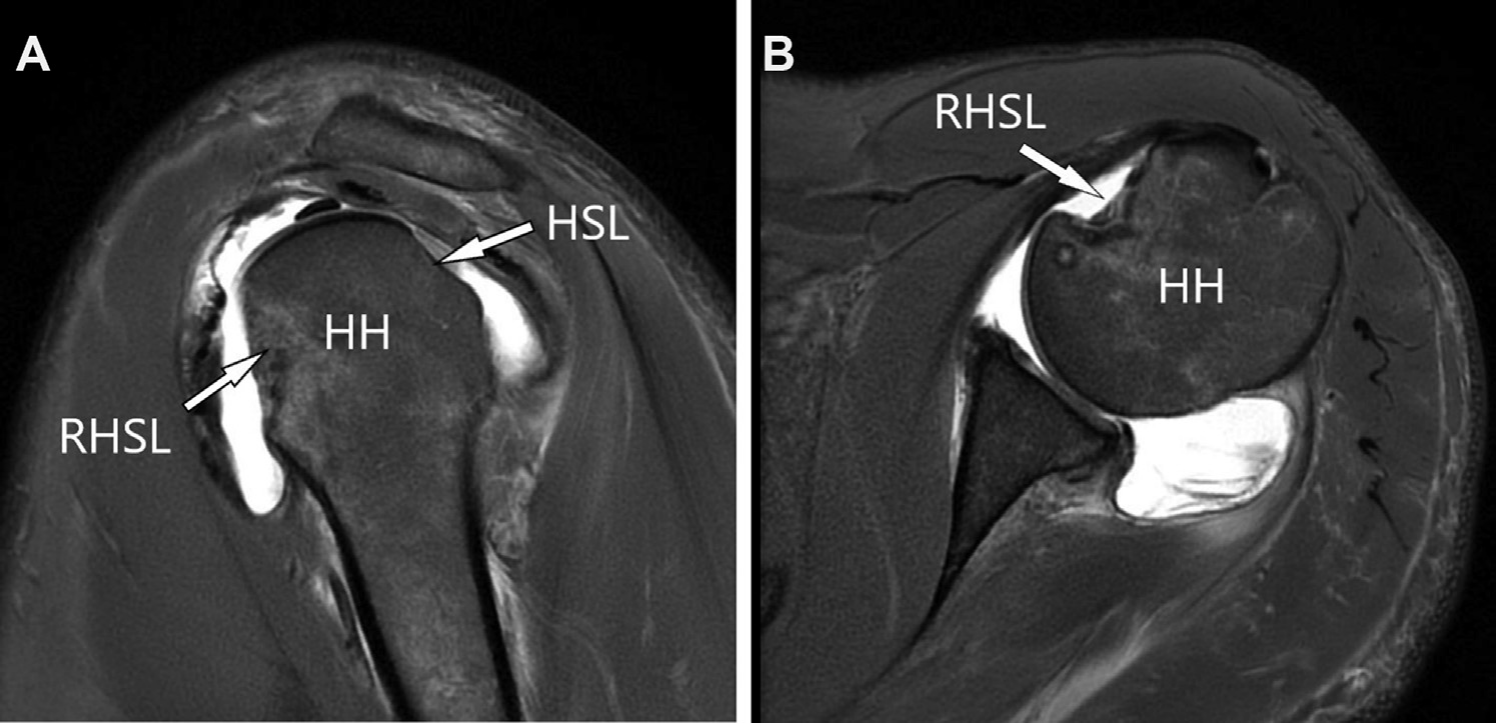
Shoulder MRI showing lesions. (A) Sagittal plane: simultaneous acute HSL and RHSL after traumatic injuries. (B) Axial plane: RHSL. There are no relevant posterior glenoid defects, and the anterior glenoid defect is <20%. Abbreviations: HH, humeral head; HSL, Hill-Sachs lesion; MRI, magnetic resonance imaging; RHSL, reverse Hill-Sachs lesion.

Our technique (Video 1) uses the traditional lateral decubitus position. A 30° arthroscope is used during the whole procedure. A standard posterior portal is established, and a thorough diagnostic arthroscopy is performed to identify all intra-articular pathology. Patients with HSL and RHSL (Fig 1) often also have anterior and posterior labral lesions. Three portals are required: the superior viewing portal without a cannula, an anterior portal with an 8.75-mm threaded cannula (Arthrex, Naples, FL), and a posterior portal with a 6-mm threaded cannula (Arthrex) (Table 1).

**Table 1:** Pearls and pitfalls

Pearls	Pitfalls
Acute surgery facilitates the reduction of labral lesions and avoids potential joint deterioration secondary to the great anteroposterior instability.	Caution must be taken when debriding anterior to the subscapularis tendon to avoid the axillary nerve at the inferior border of the tendon.
Diagnostic arthroscopy is initially performed to rule out possible associated injuries. The use of arthroscopic cannulas (8.5 mm anterior and 6 mm posterior) avoids interposition of soft tissue with the sutures	If insufficient rotator interval tissue is debrided, it is difficult to fully visualize the anterior, extra-articular portion of the subscapularis tendon.
The superior viewing portal allows access to and repair of the anterior and posterior labral lesions and non-anatomical techniques of filling the bony defects of the humeral head with the infraspinatus (remplissage) and subscapularis (modified McLaughlin).	The posterior labrum must be treated first, otherwise it will be much more difficult to correct after RHSL filling.
Anchorages with double suture loading allow better reconstructions with fewer anchorages.	In the RHSL, the anchors must be placed at the center of the defect to avoid excessive loss of external rotation.
The number of sutures and suture anchors used for “subscapularis remplissage” should be based on size of the lesion.	Do not pass sutures through the subscapularis tendon too medially to avoid excessively tensioning the tendon
The sequence of reduction and knotting (posterior labrum - prepare remplissage - anterior labrum - knot remplissage - Mc Laughlin) and the orderly management of the sutures avoids complications when there is a large number of implants.	

**Figure 2:**
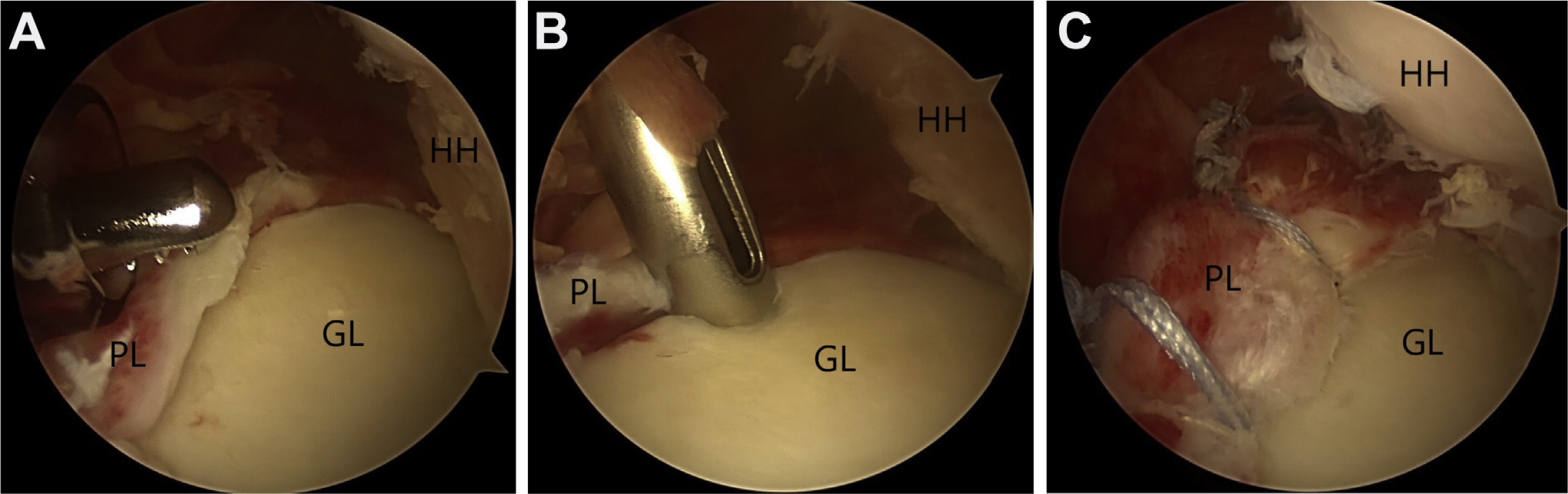
(A) PL lesion. (B) Posterior labral repair, with 3 × 3-mm double suture-loaded anchors (Biosuturetak), passing the tissue with right Suturelasso and knotting the posterior labral repair (C). Abbreviations: GL, glenoid; HH, humeral head; PL, posterior labral.

The procedure begins with the posterior labrum repair. After debridement and mobilization of the posterior labral tear, multiple 3-mm double suture-loaded anchors (Fig 2) (Biosuturetak; Arthrex) are placed along the posterior glenoid rim, and the sutures are sequentially passed, with Suturelasso, and tied to reapproximate the labrum and retension the posterior capsuloligamentous structures.

**Figure 3:**
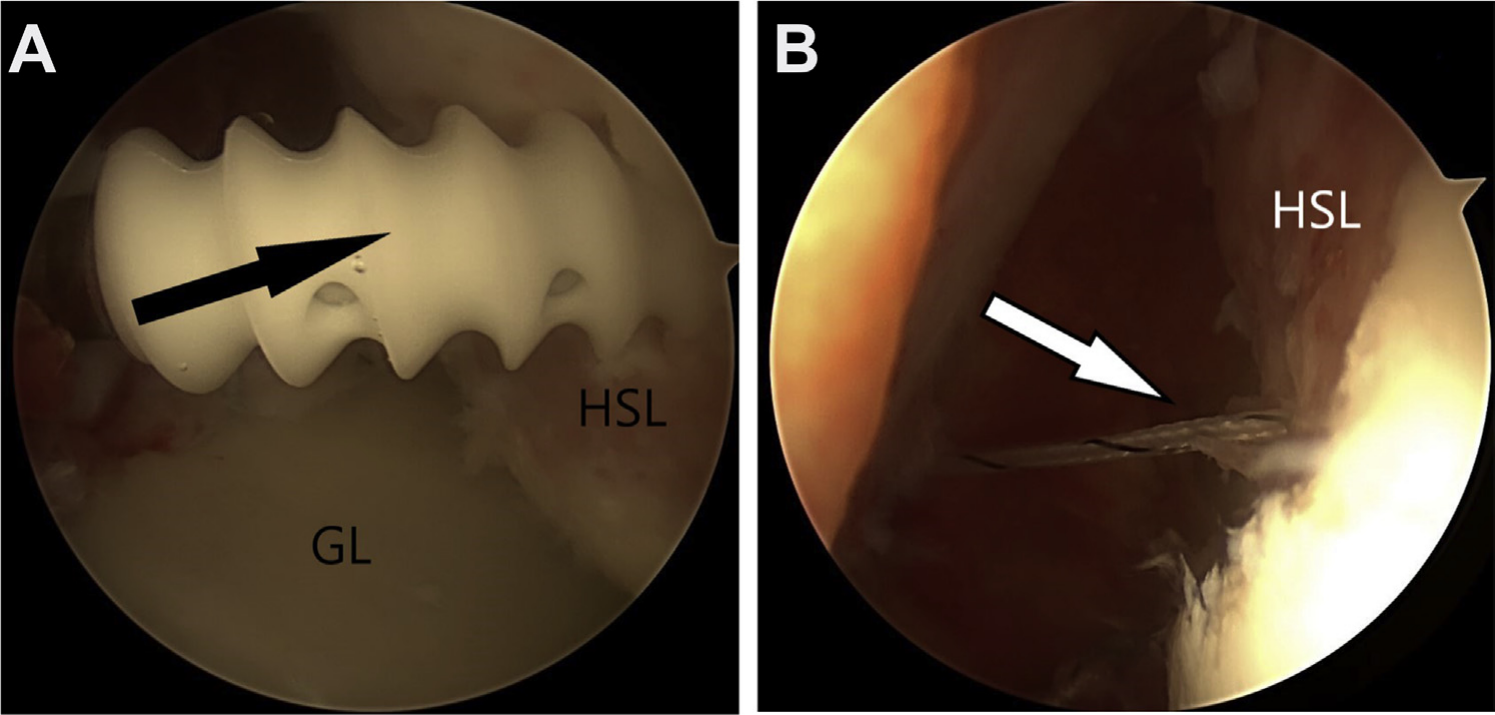
5-mm resorbable threaded implant (A) (Bio-Corkscrew) is prepared for remplissage, but without passing the sutures through the tissue (B). The implant is placed in the part closest to the cartilage of the Hill-Sachs defect.

Second, to treat the HSL, a 5-mm resorbable threaded implant (Bio-Corkscrew; Arthrex) is prepared for remplissage, but without passing the sutures through the tissue. The implant is placed in the part closest to the cartilage of the Hill-Sachs defect (Fig 3). The anchor is placed at this time because if it is placed after repairing the anterior labrum, it could damage the repair. In addition, traction on the sutures in the anterior labral repair phase is a maneuver that reduces the humeral head and facilitates repair of the anterior labrum.

**Figure 4:**
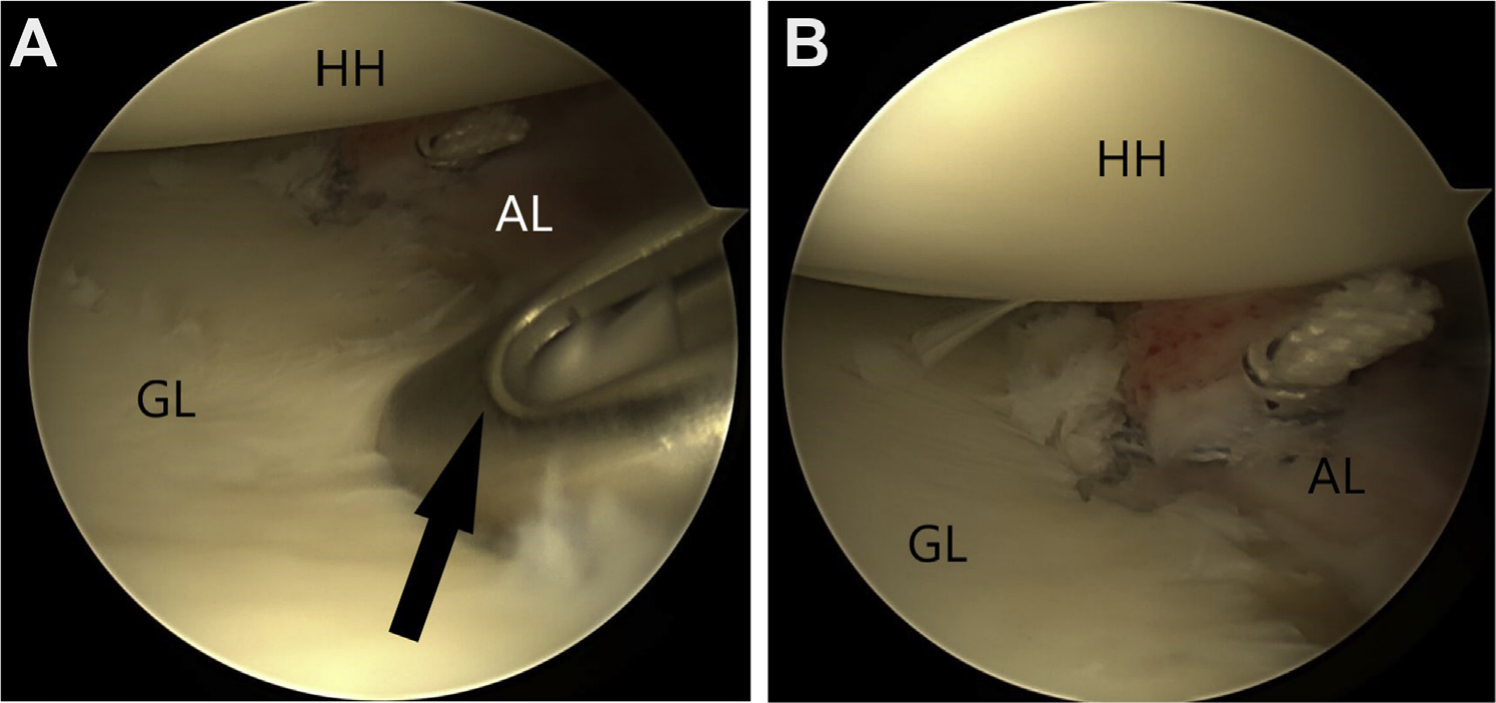
Detachment of the anterior labrum (AL) is performed with a periostotome to lift it up to the underside of the glenoid (A). After checking that the anterior labrum can be mobilized and reduced to its anatomic area, the anterior labrum is repaired (B) with multiple 3-mm double suture-loaded anchors (Biosuturetak), passing the tissue with Suturelasso.

**Figure 5:**
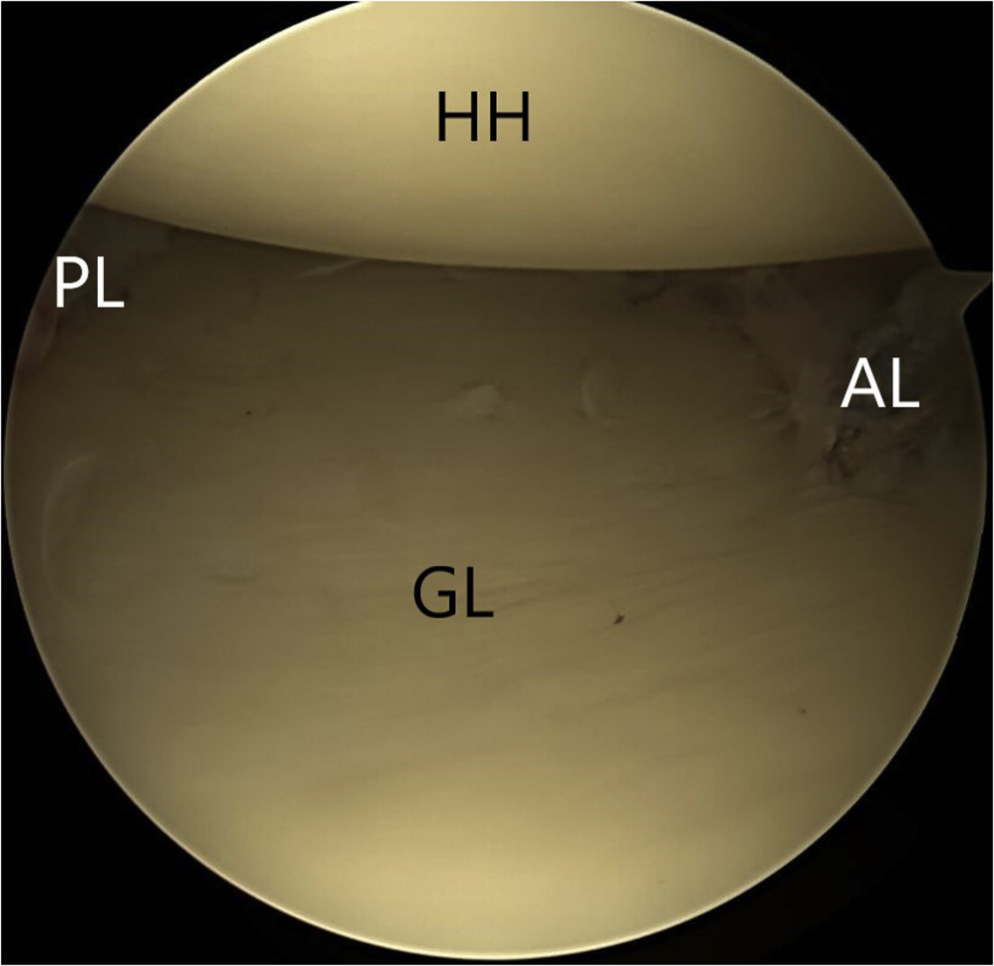
The head is reduced and centered on the glenoid.

Third, detachment of the anterior labrum is performed with a periostotome to lift it to the underside of the glenoid (Fig 4). After checking that the anterior labrum can be mobilized and reduced to its anatomic area, the anterior labrum is repaired with multiple 3-mm double suture-loaded anchors (Biosuturetak), passing the tissue with Suturelasso (Fig 4). The anterior labral repair is tied. The head is now reduced and centered on the glenoid (Fig 5).

**Figure 6:**
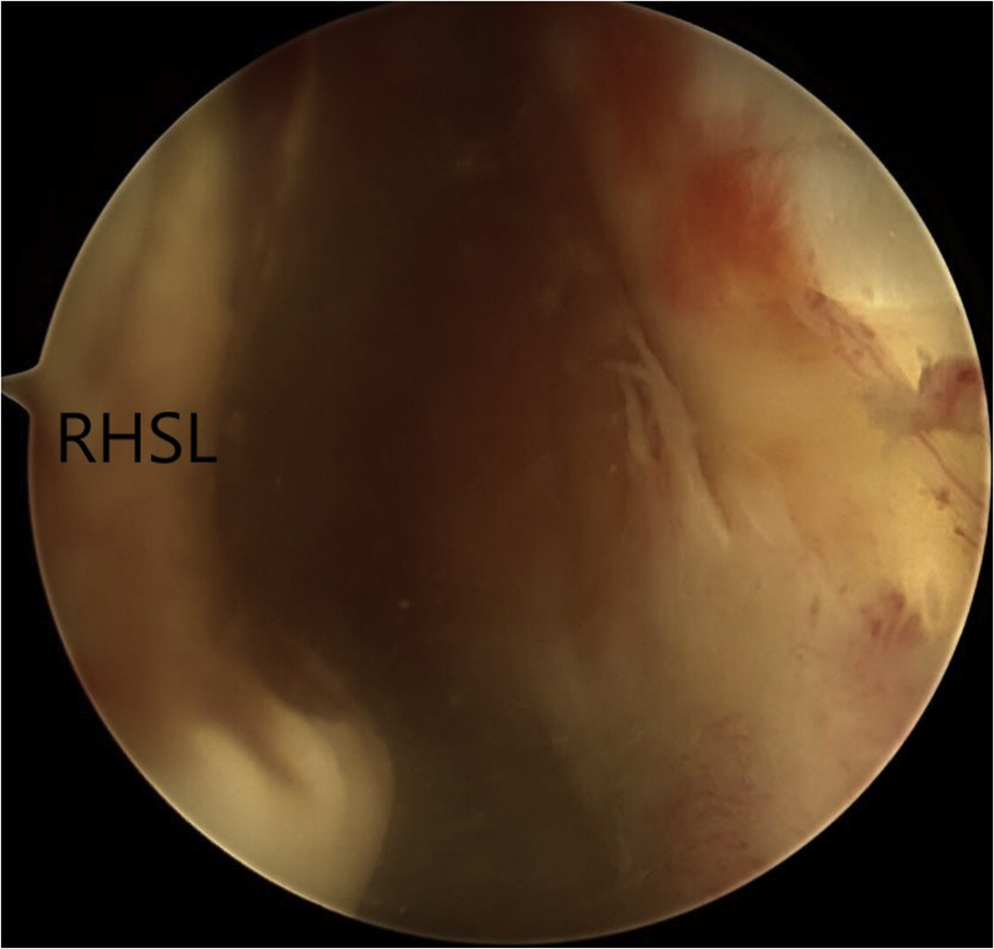
The reverse Hill-Sachs lesion is identified and debrided of all soft tissue using an arthroscopic shaver.

**Figure 7:**
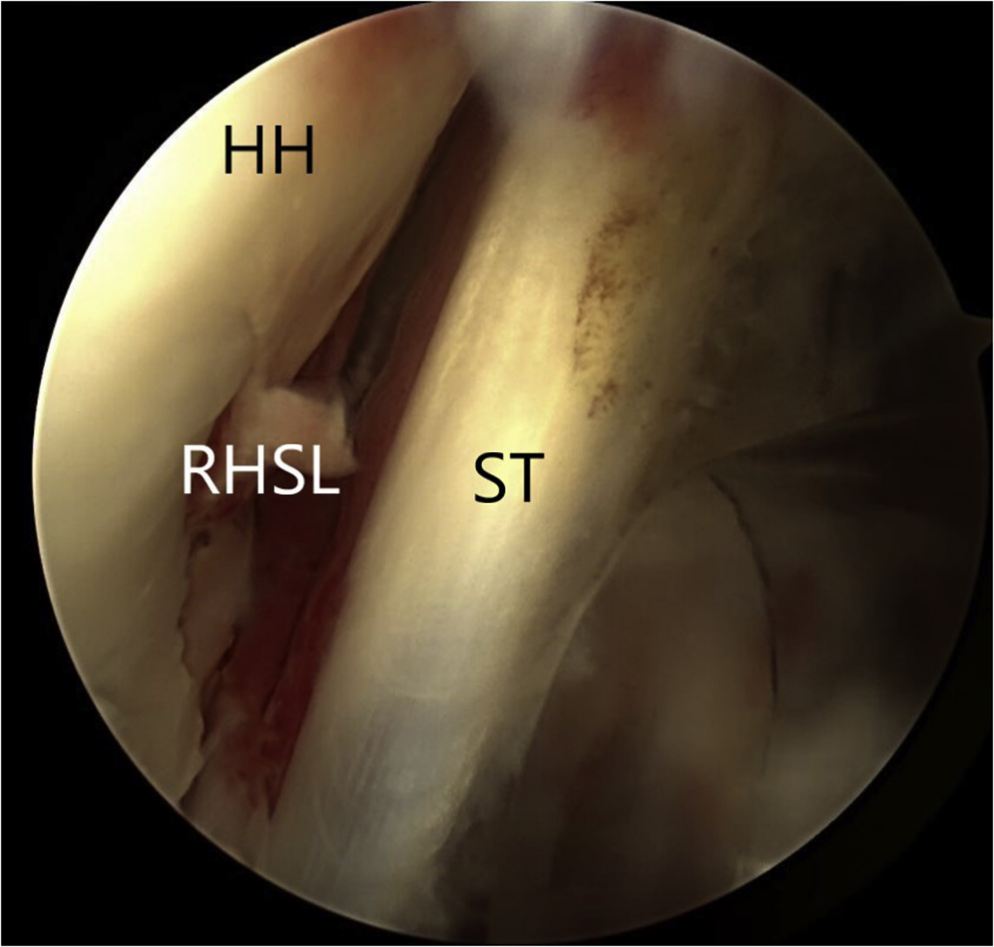
The arthroscopic shaver is also used to excise the central portion of the rotator interval tissue to generate better exposure of the subscapularis tendon (ST).

**Figure 8:**
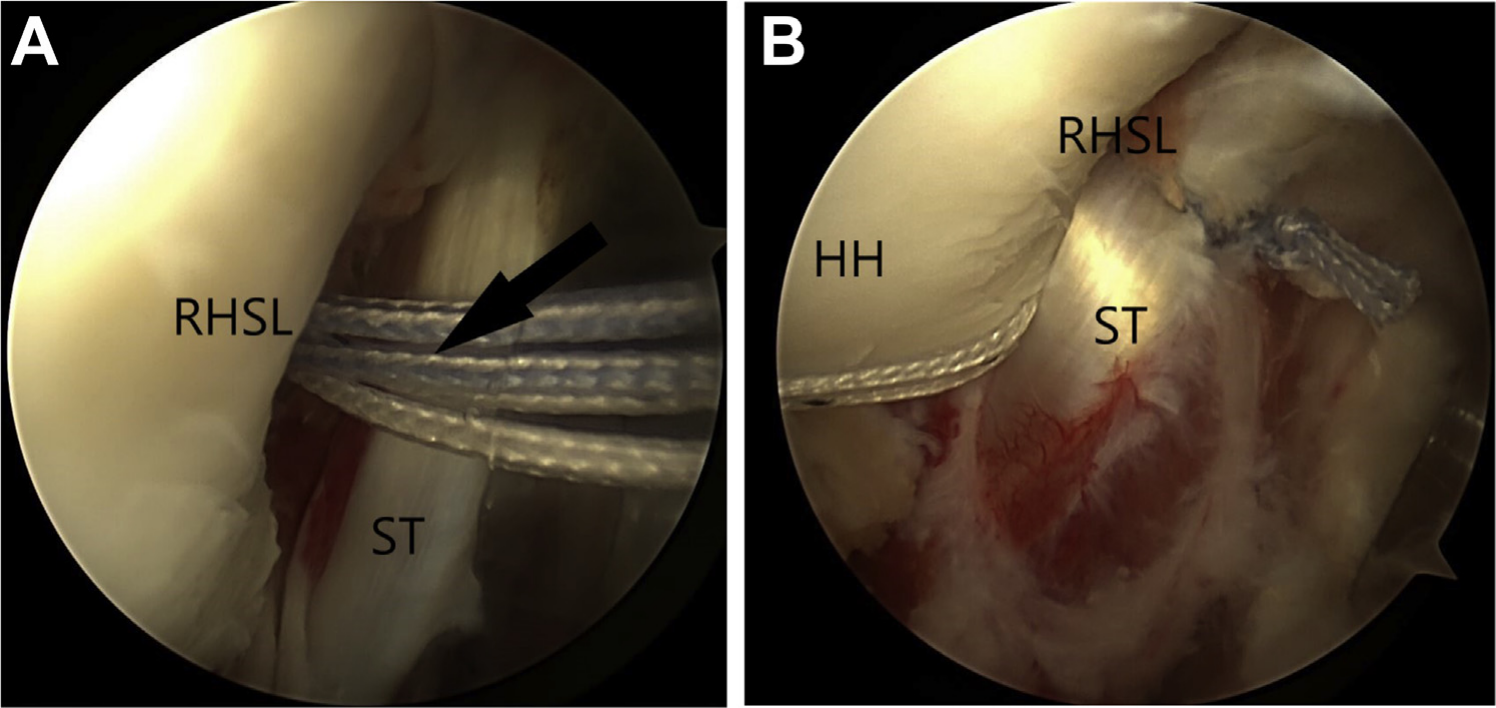
The center of the reverse Hill-Sachs lesion is identified (A), and a 5-mm resorbable threaded implant (Bio-Corkscrew) is inserted from the anterior cannula. The tissue is passed through the top of the subscapularis with suture lasso, with mattress stitches (B).

Fourth, the modified McLaughlin procedure is performed. The reverse Hill-Sachs lesion is identified and debrided of all soft tissue using an arthroscopic shaver (Fig 6). The arthroscopic shaver is also used to excise the central portion of the rotator interval tissue to generate better exposure of the subscapularis tendon (Fig 7). This facilitates visualization of the anterior surface of the subscapularis tendon and suture passage across the subscapularis tendon. The center of the RHSL is identified, and a 5-mm resorbable threaded implant (Bio-Corkscrew) is inserted from the anterior cannula, and the tissue is passed through the top of the subscapularis with suture lasso, with mattress stitches (Fig 8A). The soft tissue is knotted, and the subscapularis tendon is thus advanced and secured into the RHSL (Fig 8B).

**Figure 9:**
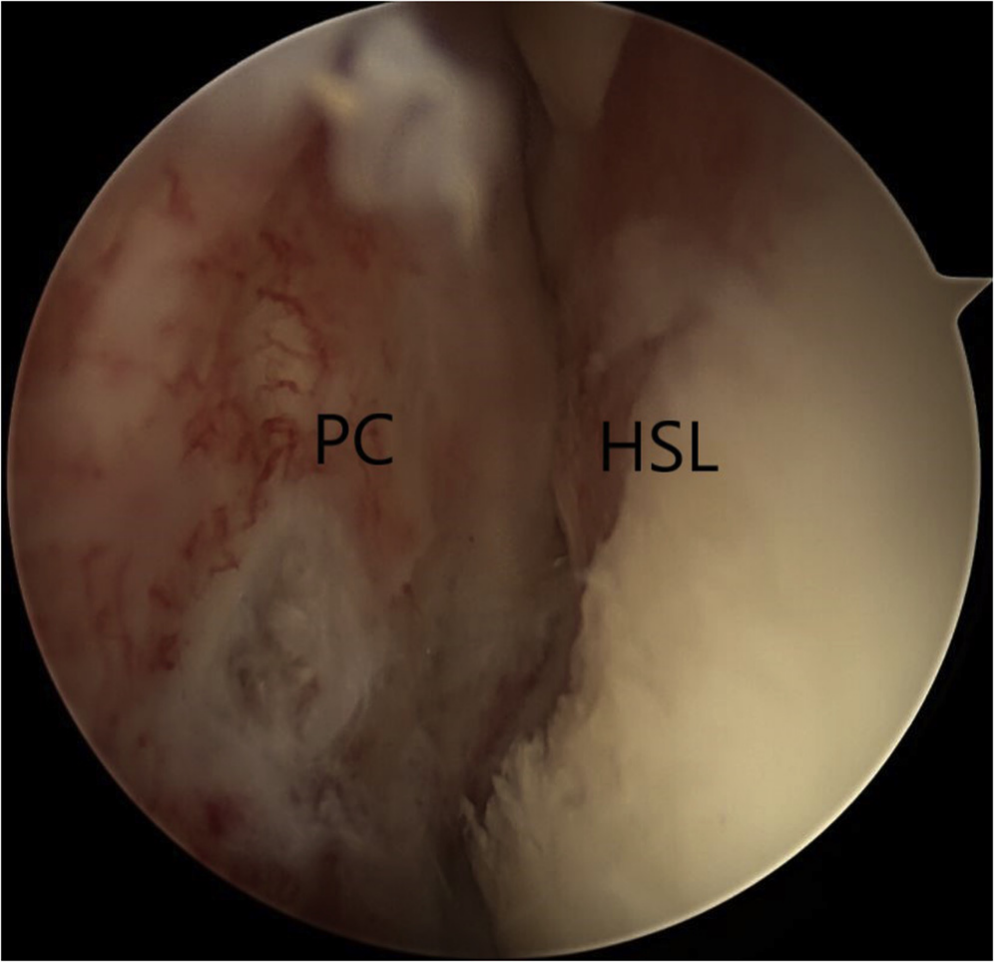
The posterior threaded cannula is partially removed until it is extra-articular (behind the infraspinatus) but deep to the deltoid. Using straight through forceps (Birdbeak; Arthrex), the infraspinatus and posterior capsule (PC) are traversed inferiorly to superiorly at 5-mm intervals, retrieving each of the 2 implant sutures (Bio-Corkscrew). The remplissage is tied, achieving a good filling of the Hill-Sachs defect with the infraspinatus and posterior capsule (tenodesis effect).

Finally, the remplissage technique is completed. For this, the posterior threaded cannula is partially removed until it is extra-articular (behind the infraspinatus) but deep to the deltoid. Using straight through forceps (Birdbeak; Arthrex), the infraspinatus and posterior capsule are traversed inferiorly to superiorly at 5-mm intervals, retrieving each of the 2 implant sutures (Bio-Corkscrew). The remplissage is tied, achieving a good filling of the Hill-Sachs defect with the infraspinatus and posterior capsule (tenodesis effect) (Fig 9).

## Rehabilitation

The patient must wear a sling for 3 weeks. During this period, the sling may be removed 4 or 5 times a day for pendulum and passive mobility exercises. Three weeks after surgery, the patient begins rehabilitation. This begins with joint range exercises according to pain tolerance and complementary antalgic and anti-inflammatory measures in an attempt to recover functional joint ranges by week 6, without forcing external rotation. Subsequently, muscle work is started from week 8 with eccentric exercises with elastic bands in a progressive manner.

## Discussion

Hill-Sachs lesions can be present from the initial dislocation event as well, and may go unrecognized; this can alter the necessary bony constraint within the glenohumeral joint.^11^ To deal with HSLs, remplissage is a safe procedure with low complication rates, low recurrent instability rates, good patient outcome scores, and minimal loss of range of motion compared with many of the alternative techniques.^4^

On the other hand, a great number of techniques have been described to treat RHSLs.^5^ Historically, most of the current treatments for posterior shoulder instability were based on the humeral head defect size. However, consensus on a specific treatment is yet to be established.^12^ In 1952, McLaughlin^6^ described a technique involving the transfer of the subscapularis tendon into the anterior humeral defect for the first time. Thereafter, several authors modified the procedure by transferring the lesser tuberosity along with the subscapularis tendon instead of transferring the tendon on its own, with some variations,^13^ or using the subscapularis tendon to fill the defect without detaching the tendon.^14^ On the other hand, disimpaction of the impression fracture and bone grafting with an iliac crest graft or an allograft have been described.^15^

A few years ago, Krackhardt et al.^7^ proposed the first all-arthroscopic technique by fixing the subscapularis tendon in the humeral impression fracture using 2 suture anchors placed medially and laterally in the RHSL; after that, the wires were crossed through the subscapularis tendon and then knotted, commonly known as the reverse remplissage technique. Since then, variations in location and number of suture anchors, suture configuration, or use of the medial glenohumeral ligament instead of the subscapularis tendon have been proposed.^2^^,^^5^^,^^8^, ^9^, ^10^^,^^16^ The technical note we present uses portals, equipment, and suture management methods that are very familiar to the arthroscopic shoulder surgeon and can be reliably performed.

In conclusion, we present an arthroscopic treatment guideline for patients with shoulder instability due to anterior and posterior labral lesions, HSL and RHSL. We showing step by step the factors that must be taken into account to approach this surgery with the best guarantees.

## Supplementary Data

ICMJE author disclosure forms

Video 1 Technique.
